# Age-At-Injury Influences the Glial Response to Traumatic Brain Injury in the Cortex of Male Juvenile Rats

**DOI:** 10.3389/fneur.2021.804139

**Published:** 2022-01-17

**Authors:** Tabitha R. F. Green, Sean M. Murphy, J. Bryce Ortiz, Rachel K. Rowe

**Affiliations:** ^1^Department of Child Health, University of Arizona College of Medicine-Phoenix, Phoenix, AZ, United States; ^2^Phoenix Veterans Affairs (VA) Health Care System, Phoenix, AZ, United States; ^3^Department of Integrative Physiology, University of Colorado, Boulder, CO, United States; ^4^BARROW Neurological Institute at Phoenix Children's Hospital, Phoenix, AZ, United States

**Keywords:** concussion, pediatric, juvenile, inflammation, microglia, astrocyte, aging

## Abstract

Few translational studies have examined how age-at-injury affects the glial response to traumatic brain injury (TBI). We hypothesized that rats injured at post-natal day (PND) 17 would exhibit a greater glial response, that would persist into early adulthood, compared to rats injured at PND35. PND17 and PND35 rats (*n* = 75) received a mild to moderate midline fluid percussion injury or sham surgery. In three cortical regions [peri-injury, primary somatosensory barrel field (S1BF), perirhinal], we investigated the glial response relative to age-at-injury (PND17 or PND35), time post-injury (2 hours, 1 day, 7 days, 25 days, or 43 days), and post-natal age, such that rats injured at PND17 or PND35 were compared at the same post-natal-age (e.g., PND17 + 25D post-injury = PND42; PND35 + 7D post-injury = PND42). We measured Iba1 positive microglia cells (area, perimeter) and quantified their activation status using skeletal analysis (branch length/cell, mean processes/cell, cell abundance). GFAP expression was examined using immunohistochemistry and pixel analysis. Data were analyzed using Bayesian multivariate multi-level models. Independent of age-at-injury, TBI activated microglia (shorter branches, fewer processes) in the S1BF and perirhinal cortex with more microglia in all regions compared to uninjured shams. TBI-induced microglial activation (shorter branches) was sustained in the S1BF into early adulthood (PND60). Overall, PND17 injured rats had more microglial activation in the perirhinal cortex than PND35 injured rats. Activation was not confounded by age-dependent cell size changes, and microglial cell body sizes were similar between PND17 and PND35 rats. There were no differences in astrocyte GFAP expression. Increased microglial activation in PND17 brain-injured rats suggests that TBI upregulates the glial response at discrete stages of development. Age-at-injury and aging with an injury are translationally important because experiencing a TBI at an early age may trigger an exaggerated glial response.

## Introduction

Toddlers (0–4 years) and adolescents (15–18 years) are vulnerable subgroups of the population in which the incidence of traumatic brain injury (TBI) peaks (1). Higher prevalence of TBIs in these age groups is primarily associated with participation in sports (2), car accidents, domestic violence (3, 4), and falls (5). Understanding the cellular response to injury in an age-specific manner is important to enable effective patient care and personalized medicine. Little is known about the specific glial response to TBI acquired as a toddler or an adolescent, which are two unique time periods for brain development. Support exists for these developmental periods being windows of both neuroprotection and increased vulnerability, phenomena that are likely mutually exclusive (6–8).

TBI results from mechanical forces applied to the brain, which can cause contusion, hemorrhage, diffuse axonal injury, and shearing (9). TBI triggers neuroinflammatory cascades that result in cellular damage and functional deficits. Neuroinflammation is mediated by microglia and astrocytes that change their morphology and transcriptional profile in response to injury (10). Acutely, microglia-mediated inflammation is beneficial and clears damaged cells and contents associated with TBI (11). Activation of microglia in juveniles following experimental TBI may also be important for the removal of dying neurons (12). However, long-term persistence of glial activation and cytokine release causes a self-perpetuating state of chronic inflammation, exacerbates the brain injury, and can lead to neuronal damage and neurodegeneration (11, 13). Microglia morphology is altered in response to injury (14). Activated microglia undergo a continuum of morphological transitions from a highly branched phenotype of surveying microglia to a rounded phagocytic morphology characterized by an enlarged cell body and retracted processes (14, 15). Microglial morphology can be assessed by immunohistochemistry using ionized calcium binding protein adapter molecule 1 (Iba1). Astrocytes also take on a hypertrophic phenotype when activated and increase their expression of glial fibrillary acidic protein (GFAP).

Herein, we used morphological changes in both microglia and astrocytes as physical indicators of distress, damage, and/or inflammation in the cortex of the brain, whereby reduced ramification was an indicator of microglial activation. Few pre-clinical models have examined TBI-associated pathology in juvenile rats (8). Therefore, we used a comprehensive time course after experimental TBI in rats to examine the acute and sub-acute cortical glial response (microglial activation and GFAP expression) to TBI. We used juvenile rats subjected to TBI at post-natal day (PND) 17, which models early childhood in humans, or PND35, which models adolescence in humans (13, 16).

We chose to investigate the glial response as a function of age-at-injury because of mounting evidence that indicates a differential immune response to injury throughout the lifespan (17, 18). During early life, the brain is undergoing vast circuit remodeling and is particularly vulnerable to injury and inflammation. As such, TBI incurred at a younger age may elicit a different inflammatory outcome compared to a brain injury at an older age. Previous studies have shown that different inflammatory signaling pathways are prevalent in the juvenile rodent brain when compared to adults (18, 19). Furthermore, younger animals experience a greater infiltration of leukocytes after TBI (20), which may contribute to the blood brain barrier breakdown observed in juvenile rats (17, 21). Infiltrating leukocytes can increase the number of inflammatory cells and subsequently elevate cytokine levels, free radical production, and protease release (22). Together, these secondary injury processes contribute to tissue damage and microglial activation. Based on the age-dependent dimorphism in inflammatory signaling, we hypothesized that rats injured at PND17 would exhibit a greater cortical glial response, which would persist into early adulthood, compared to rats injured at PND35.

## Methods

### Study Design

**Figure 1:**
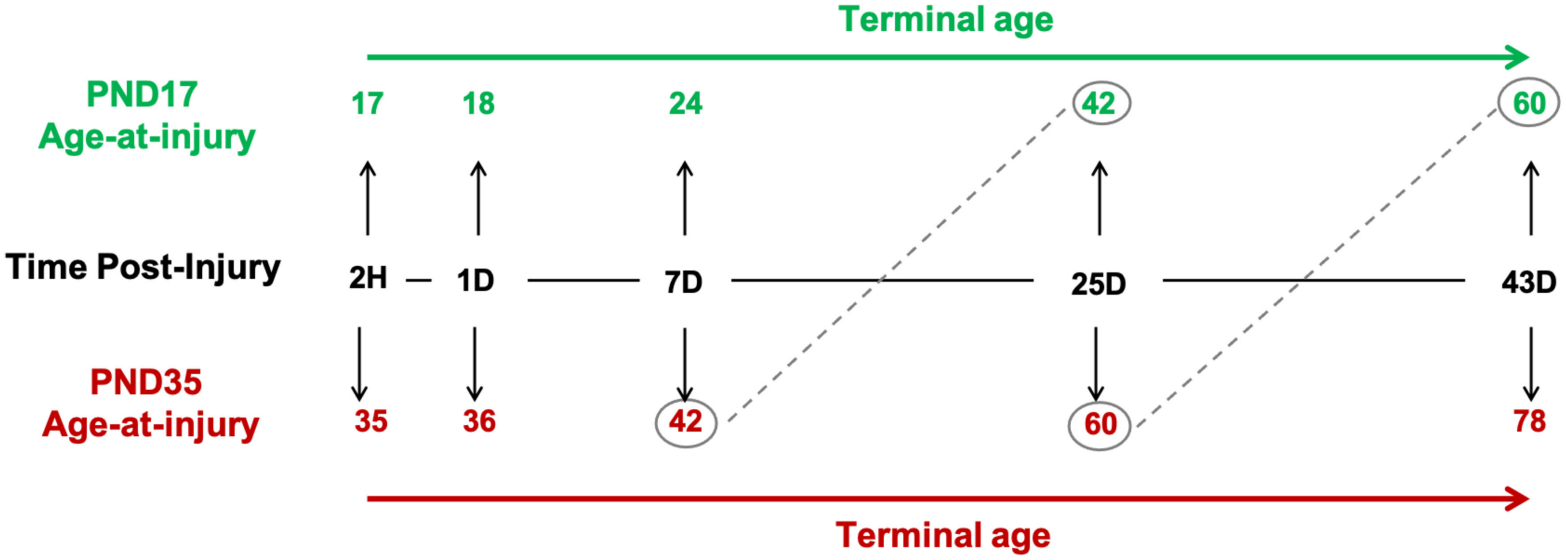
Study design. Post-natal day (PND) 17 and PND35 rats (*n* = 75) received midline fluid percussion injury or sham surgery. In three cortical regions (peri-injury, S1BF, perirhinal), we investigated the glial response relative to age-at-injury (PND17/PND35), time post-injury (2H, 1D, 7D, 25D, and 43D), and post-natal-age, such that rats injured at PND17 or PND35 were compared at the same post-natal-age (e.g., PND17 + 25D post-injury = PND42; PND35 + 7D post-injury = PND42).

We investigated the glial response relative to age-at-injury (PND17 or PND35), time post-injury (tissue collection: 2H, 1D, 7D, 25D, and 43D), and post-natal age, such that rats injured at PND17 or PND35 were compared at the same post-natal age (e.g., PND17 + 25D post-injury = PND42; PND35 + 7D post-injury = PND42; Figure 1).

### Rigor

All experiments were performed according to the National Institutes of Health and Institutional Animal Care and Use Committee (IACUC) guidelines. The Animal Research: Reporting *in vivo* Experiments (ARRIVE) guidelines were followed. For data analyses, a total of 75 rats were used (sham *n* = 31, TBI *n* = 44). Exclusion criteria were predetermined such that rats that lost >20% of their body weight or had unmanageable pain were excluded; however, no rats in the study met these criteria and, therefore, none were excluded post-TBI. Pre-determined inclusion criteria included a righting reflex time >180 seconds and no breach of the dura during surgery. All samples and files were re-labeled with codenames by an investigator not associated with the current study to ensure that all experiments were conducted in blinded conditions. The group sizes for this study were: PND17 2H TBI *n* = 4, PND17 24H TBI *n* = 5, PND17 24H sham *n* = 5, PND17 7D TBI *n* = 6, PND17 7D sham *n* = 4, PND17 25D TBI *n* = 5, PND17 25D sham *n* = 4, PND17 43D TBI *n* = 5, PND17 43D sham *n* = 5, PND35 2H TBI *n* = 4, PND35 24H TBI *n* = 5, PND35 24H sham *n* = 5, PND35 7D TBI *n* = 5, PND35 7D sham *n* = 4, PND35 25D TBI *n* = 5, PND35 25D sham *n* = 4.

### Animals

Male Sprague Dawley rats (Envigo, Indianapolis, IN) were used for all experiments. Rats were housed in a 12 h light: 12 h dark cycle at a constant temperature (23°C ± 2°C) with food and water available *ad libitum* according to the Association for Assessment and Accreditation of Laboratory Animal Care International guidelines. All rats were acclimated from shipping a minimum of one week prior to experiments. PND17 rats were shipped with the dam. After surgery, post-operative care via physical examination took place to monitor each animal's condition. PND17 rats were returned to their dam following surgery and midline fluid percussion injury (mFPI) until tissue collection (2H, 24H, 7D), or until they were weaned at PND24. Average PND17 pre-surgical weight was 28.2 ± 3.2 g. Average PND35 pre-surgical weight was 135.5 ± 14.3 g. Weights and health conditions were monitored and documented throughout the experiment. Animal care and experiments were approved by the Institutional Animal Care and Use Committee (IACUC) at the University of Arizona (protocol 13–460).

### Midline Fluid Percussion Injury

For surgery, all rats were administered 5% isoflurane in 100% oxygen for 5 min and then secured in a stereotaxic frame. Anesthetization was maintained with continuous isoflurane delivery at 2.5% via nosecone. A midline incision was made and a craniectomy (outer diameter 3 mm in PND17 rats and 4 mm in PND35 rats) was trephined midway between bregma and lambda (23). The skull flap was then removed with care not to disrupt the dura or superior sagittal sinus underlying the craniectomy site. An injury hub (prepared from the female portion of a Luer-Loc needle hub) was fixed over the craniectomy using cyanoacrylate gel and methyl-methacrylate (Hygenic Corp., Akron, OH). Post-surgery, rats were placed on a heating pad and monitored until ambulatory.

Approximately 60–120 min after surgery, rats were subjected to mFPI with methods we have previously described for PND17 and PND35 rats (13, 23, 24). Rats were re-anesthetized with 5% isoflurane in 100% oxygen delivered for 3 min. The hub assembly on the skull was filled with saline and attached to the FPI device (custom design and fabrication, Virginia Commonwealth University, Richmond, VA). When a toe pinch withdrawal response was detected, the pendulum was released causing a fluid pulse directly onto the dura resulting in a mild to moderate brain injury in all rats [PND17 = 1.5 atmospheres pressure (atm), PND35 = 1.9 atm] (13, 23, 24). Sham rats were connected to the device, but the pendulum was not released. Hubs were removed immediately after injury or sham injury and rats were monitored for apnea, righting reflex time (time from the initial impact until the rat spontaneously righted itself from a supine position), and a fencing response (25). After rats spontaneously righted, brains were inspected for herniation, hematomas, and integrity of the dura. Brain-injured rats included in this study had an average righting reflex time of 318 s, indicative of a mild to moderate injury (23, 24), and had no disruption to the underlying dura. Sham rats spontaneously righted (~20 s) when removed from the device. Rats were re-anesthetized and scalp incisions were cleaned with sterile saline and closed. Rats were placed in a heated recovery cage and monitored until ambulatory. Rat welfare was evaluated and documented daily during post-operative care via physical examination.

### Cryoprotection and Tissue Sectioning

At pre-determined time points post-injury (2H, 1D, 7D, 25D, and 43D), a lethal dose of Euthasol^®^ was administered. Rats underwent transcardial perfusion with 4% paraformaldehyde (PFA) after flushing vasculature with phosphate buffered saline (1 × PBS). From the time of tissue harvest, tissue samples were treated identically throughout the experiment to reduce variation. Brains were harvested from the skull and drop fixed in 4% PFA for 24 h. Brains were cryoprotected by successive incubation in 15 and 30% sucrose, each for 24 h. Brains were then removed from sucrose and the left hemisphere from each animal was frozen in groups of 6–9 using the Megabrain technique as previously published (26). Megabrains were cryosectioned in the coronal plane at 40 μm and mounted on superfrost slides and stored at −80 °C. Sections were removed from the freezer and baked at 56°C for 3 h prior to undergoing immunohistochemistry.

### Immunohistochemistry and Analysis

Each immunohistochemistry stain was performed on 4 randomly selected brain slices located between bregma and lambda from the left hemisphere of each animal (total area of 160 μm), and 3 regions of interest [peri-injury, primary somatosensory barrel field (S1BF), perirhinal] per slice were analyzed. Based on our findings of the biomechanical mechanism of mFPI (27), and our previously published work on TBI-induced neuropathology in juvenile rats (13), we chose three cortical regions of interest for the current study. We selected two cortical areas that, based on our previous research, exhibit extensive pathology after mFPI (peri-injury and S1BF), as well as a remote cortical region for comparison. Regions were selected using visual anatomical landmarks while the microscope was out of focus to allow accurate selection of brain region without sampling bias.

Iba1: To analyze microglia morphology, brains were stained for ionized calcium binding protein adapter molecule 1 (Iba1). To improve scientific rigor, Iba1 staining was performed in a single round of staining to minimize variance and allow comparisons. Slides were rehydrated in 1 × PBS after baking (3 h). Antigen retrieval was performed using sodium citrate buffer (pH 6.0). Slides were then washed in 1 × PBS. Hydrophobic barrier pen was applied to the perimeter of the slide and slides were placed in a humidity chamber. Blocking solution was immediately applied [4% normal horse serum (NHS), 0.1% Triton-100 in 1 × PBS] with an incubation time of 60 min. Following blocking, primary antibody solution (rabbit anti-Iba1; WAKO cat #019919741; RRID: AB_839504; at 1:1000 concentration in 1% NHS, 0.1% triton-100 in 1 × PBS) was applied and left to bind overnight at 4°C. Slides were then washed in 1 × PBS + 0.1% tween-20. Secondary antibody solution [biotinylated horse anti-rabbit IgG (H + L); vector BA-1100; RRID: AB_2336201; at 1:250 concentration in 4% NHS and 0.4% triton-100 in 1 × PBS] was applied and incubated for 60 min. Slides were washed in 1 × PBS + 0.1% tween-20. Endogenous peroxidases were blocked in 200 ml 1 × PBS + 8 ml H_2_O_2_ for 30 min. After washing in 1 × PBS + 0.1% tween-20, Avidin-Biotin Complex (ABC) solution (Vectastain ABC kit PK-6100) was applied and incubated for 30 min. Slides were washed in PBS + 0.1% tween-20 and then 3,3′-diam-inobenzidine (DAB) solution (from Vector DAB peroxidase substrate kit SK-4100) was applied and incubated for 10 min and, following this, slides were immediately placed in water. Tissue was dehydrated in ethanol (70, 90, and 100%) and cleared with citrosolve. Coverslips, matching microscope specifications, were applied using dibutylphthalate polystyrene xylene mounting medium.

GFAP: To analyze astrocytes, brains were stained for glial fibrillary acidic protein (GFAP). An identical protocol was followed to that described for Iba1, using solutions; blocking = 5% NHS, 0.1% triton X-100 in 1 × PBS; primary antibody solution = polyclonal rabbit anti-glial fibrillary acidic protein #Z0334; RRID: AB_10013382; at a 1:1000 concentration in 2% NHS and 1 × PBS solution; secondary solution = biotinylated horse anti-rabbit IgG (H +L); vector BA-1100 at 1:250 concentration in 4% NHS and 0.4% triton-100 in 1 × PBS. The DAB incubation time was 5 min.

**Figure 2:**
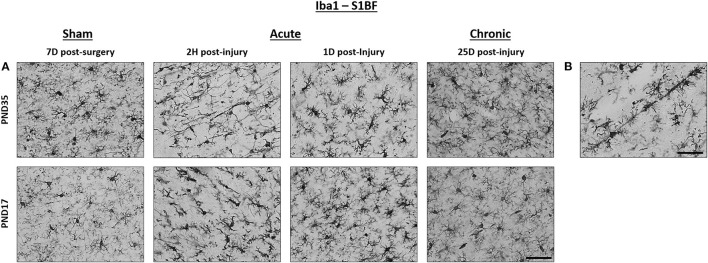
TBI activated microglia in both PND17 and PND35 rats. Iba1 stained microglia in PND17 and PND35 rats. **(A)** At 2 h (H), 1 day (D), and 25D post-injury, microglial activation was observed in PND17 and PND35 rats subjected to TBI compared to uninjured shams. All representative images are from the S1BF. **(B)** Rod microglia were observed in PND17 rats but not PND35 rats. Scale bars = 50 μm. (sham *n* = 39, TBI *n* = 35).

Imaging and analysis: Z-stack images of stained tissue were taken at 400 × (40 × objective lens, 10 × ocular lens) using Zeiss Imager A2 microscope via AxioCam MRc5 digital camera and Neurolucida 360 software, with consistent brightness, numerical aperture, and Z-stack height (Figure 2). Nyquist theorem was followed to ensure the signal adequately represented our biological samples. Iba1 staining was analyzed using the skeletal analysis plugin following the protocol previously published (28, 29). Microglial cell somas were counted manually to obtain total microglial count. Branch length and processes were recorded and divided by the number of cells in each region of interest. Microglial cell bodies were measured using the multipoint area selection tool to calculate cell body area and perimeter. Although cell body area and perimeter are typically proportional, it is possible for a cell body to have a more complex shape but not larger total area. For this reason, both measurements were included in our analyses. To capture the hypertrophic morphological changes and increased GFAP expression seen in astrocytes after injury, GFAP images were analyzed for number of GFAP + cells and average number of pixels per cell using ImageJ software. Cell somas were counted manually. The average number of pixels per cell (referred to as cell coverage henceforth) was recorded to assess gross morphological changes in GFAP^+^ astrocytes following mFPI. No alternations/settings changes were made to any images prior to analysis. All imaging analyses were performed on Z-stack images, which is the most appropriate sampling method to capture our sample.

### Statistical Analyses

Outcomes in this study were counts, percentages, and distances or areas, which we analyzed using Poisson or negative-binomial regression, beta regression, and log-normal or Gaussian regression, respectively (30–32). Integer count outcomes were cell coverage (*n* pixels/cell), cells (*n*), and mean number of branches per microglial cell (*n*); pixel coverage was the only bounded percentage outcome; and microglial branch length (μm), cell body perimeter (μm), and cell body area (μm^2^) were continuous outcomes with lower bounds truncated at zero but untruncated upper bounds. To determine whether Poisson or negative-binomial distributions were best suited for analyzing the count data, we tested for overdispersion (i.e., variance > mean) using the dispersiontest() function in the AER package (33, 34) of the R statistical computing environment (22).

For each outcome type, we fit three multivariate multi-level multiple regression models to test differences between treatment groups (sham vs. TBI) for each PND injury age (data collected from all time post-injury groups were pooled), PND terminal age, and time post-injury (35–38). In total, we fit 24 multivariate models, with each having three submodels, for a total of 72 models. The three submodels for each outcome type corresponded to the following population-level effects (*sensu* fixed effects): (1) an interaction between treatment group and time post-injury, (2) an interaction between treatment group and PND injury age, and (3) an interaction between treatment group and PND terminal age. In each model, we included group-level varying intercepts (*sensu* random effects) for surgery day to account for potential variation or dependency that may have been induced by groups of rats receiving surgery on the same day (*n* = 9 surgery days). For the cell body perimeter and area models, we also included group-level varying intercepts for animal ID, because each rat had multiple datapoints due to the multiple slides that were used to collect these data.

We fit all models in a Bayesian framework, primarily because Bayesian approaches (a) do not necessitate large sample sizes for accurate parameter estimation, (b) efficiently and effectively accommodate the hierarchical data generating processes and pseudoreplication that existed in some of our outcomes, and (c) provide intuitive interpretations that mirror the human reasoning process (39–41). For each model, we applied conservatively informative priors to model parameters and variance components, based on results of previous studies [e.g., (37, 38)], our knowledge of the study systems from preliminary studies conducted by our group, and recommendations from prior statistical research. Specifically, we applied ~Normal (0, 1) priors to population-level parameters, ~Cauchy (0, 5) priors to the variance scale parameters, and ~Cauchy (0, 2) priors to the standard deviations of group-level effects, thereby appropriately restricting those variance components' parameter spaces to positive values (42). All models were fit using the Stan computational platform (43) via the R packages rstan and brms (44–46). Four Markov chains were run for each model, with each chain having a burn-in of 2,000 iterations of the No-U-Turn Sampler extension to Hamiltonian Monte-Carlo sampling, followed by 3,000 sampling iterations (37, 38). This approach produced 12,000 total posterior samples for each model. We assessed model convergence using trace plots and estimates of the potential scale reduction factor ($\hat{R}$) and effective sample sizes (*n*_eff_). Optimal values for $\hat{R}$ and *n*_eff_ were strictly 1.00–1.01 and >1,000, respectively (47, 48). We assessed model fit using posterior predictive check plots created with the R package bayesplot, comparing 1,000 posterior predictive distribution samples to the observed data (49–51).

We based inferences on a combination of model parameter estimates (β; posterior means), their 95% credible intervals, corresponding conditional marginal effects under the posterior distributions, and posterior probabilities (*P*) and Bayes factors (*K)* (52) estimated from non-linear Bayesian hypothesis tests. Additionally, we calculated effect sizes (*d)* (53, 54) using the estimated group-specific posterior means and their pooled variances (55). The strength and magnitude of support for each effect was evaluated based on the following ranges of values for *P, K*, and *d*, as we have previously reported (37, 38) and which are detailed by (52, 56), among others. Posterior probability: Weak = 0.90–0.92; Moderate = 0.93–0.95; Strong = 0.96–0.98; Decisive/Substantial ≥0.99. Bayes factor: Weak < 3; Moderate = 3–10; Strong = 11–100; Decisive/Substantial >100. Effect size: Small = 0.10–0.49; Medium = 0.50–0.79; Large = 0.80–1.19; Very large ≥1.20. For introductions to Bayesian statistics, including the advantages of Bayesian modeling and explanations of the above metrics, we encourage readers to view the following articles (41, 57, 58).

## Results

Among count outcome variables, results of dispersion tests indicated that Poisson response distributions were appropriate for GFAP + cell coverage in all three cortical regions as well as Iba1 + mean number of branches per microglial cell in all three cortical regions (dispersion range: 0.85–1.23; *z*-score range: −1.29 to 0.94). In contrast, overdispersion existed for GFAP+ cells and Iba1 + cells in all three cortical regions (dispersion range: 2.90–622.94; *z*-score range: 1.38–5.75). Therefore, negative-binomial response distributions were specified for all cell models.

### Diffuse TBI Activated Microglia in the Peri-Injury Cortex of Rats Injured at Both PND17 and PND35

**Figure 3:**
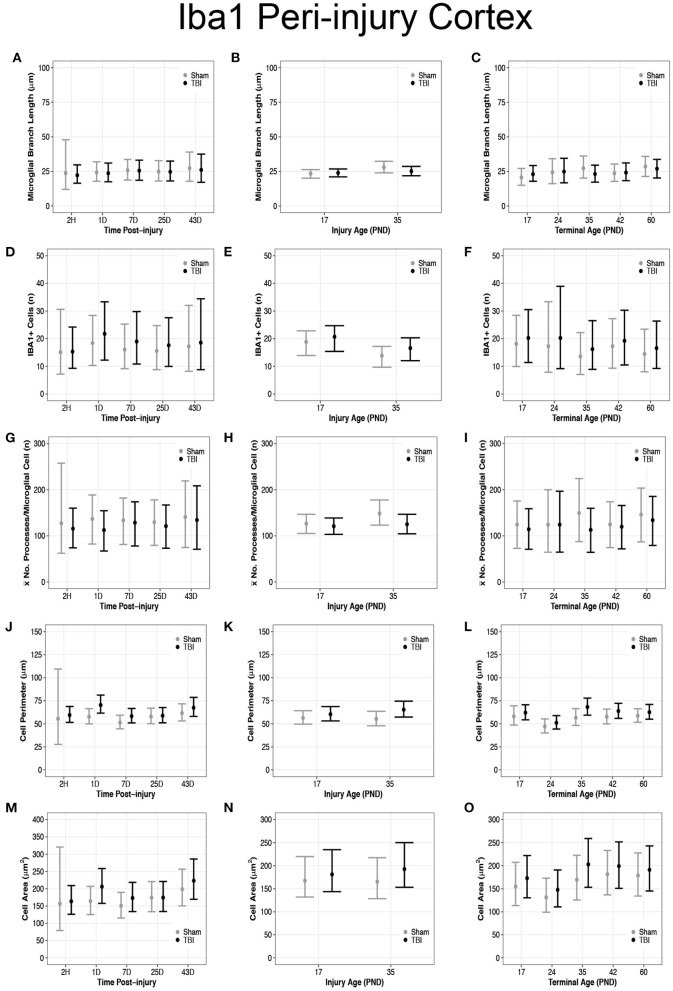
Diffuse TBI activated microglia in the peri-injury cortex of rats injured at PND17 and PND35. Point estimates (posterior means) and corresponding 95% credible intervals (highest posterior density intervals) are presented. **(A–C)** There were no differences in microglial branch lengths between sham and TBI rats across times post-injury, injury ages, or terminal ages. **(B)** PND35 shams had longer branch lengths that PND17 shams. **(D,F)** There were no differences in number of cells between sham and TBI across times post-injury and terminal ages. **(E)** Sham and TBI rats at PND35 had fewer cells than sham and TBI rats at PND17. PND17 TBI rats had more cells than PND17 shams. PND35 TBI rats had more cells than PND35 shams. **(G–I)** Sham and TBI rats had similar mean processes per microglial cell across times post-injury, injury ages, and terminal ages, **(H)** and PND35 shams had more mean processes per microglial cell than PND17 shams. **(J)** TBI rats has a longer cell perimeter at 1D, 7D, and 43D post-injury compared to shams. TBI rats at 1D had a longer cell perimeter compared to TBI at 2H, 7D, and 25D post-injury. **(K)** TBI rats had a longer cell perimeter at PND17 and PND35 compared to their respective shams. **(L)** TBI rats had a longer cell perimeter at PND35 and PND42 compared to shams and PND24 had a shorter cell perimeter compared to all other ages. **(M)** TBI rats had a larger cell area at 1D, 7D, and 43D post-injury compared to shams. TBI rats at 1D had a larger cell area compared to 2H, 7D, and 25D post-injury. **(N)** PND35 TBI rats had a larger cell area compared to PND35 shams. **(O)** Cell area was shorter at PND24 compared to all other ages.

Overall, there were more microglia in the peri-injury cortex of PND17 rats than in PND35 rats, suggesting a greater inflammatory response in the PND17 rats. There were no differences in microglial branch lengths between sham and TBI rats across times post-injury, injury ages, or terminal ages (Figures 2, 3A–C). However, PND35 shams had longer branch lengths than PND17 shams (95% CI: 0–10, *P* = 0.97, *K* = 34.40, *d* = 0.11; Figure 3B). There were no differences in number of cells between sham and TBI across times post-injury and terminal ages (Figures 3D,F). However, PND35 shams (95% CI: 0–10, *P* = 0.98, *K* = 46.62) and PND35 TBI rats (95% CI: −1 to 9, *P* = 0.94, *K* = 17.04) had fewer cells, respectively, compared to PND17 (*d* = 0.06–0.10; Figure 3E). Additionally, PND17 TBI rats had more cells than PND17 shams (95% CI: −3 to 7, *P* = 0.90, *K* = 7.96) and PND35 TBI rats had more cells than PND35 shams (95% CI: −2 to 7, *P* = 0.97, *K* = 38.6), respectively (*d* = 0.02–0.06; Figure 3E). Sham and TBI groups also had similar mean processes per microglial cell across times post-injury, injury ages, and terminal ages (Figures 3G–I). However, PND35 shams had more mean processes per microglial cell than PND17 shams (95% CI:−9–54, *P* = 0.93, *K* = 12.42, *d* = 0.01; Figure 3H).

### Diffuse TBI Increased Microglial Cell Perimeter and Cell Area Size in the Peri-Injury Cortex of Rats Injured at Both PND17 and PND35

Overall, microglia in the peri-injury cortex of PND35 rats had a greater cell body perimeter than in the peri-injury cortex of PND17 rats, suggesting a greater phagocytic potential in PND35 rats. Our data support that TBI increased cell perimeter (Figures 3J–L) and cell area (Figures 3M–O). Across times post-injury, TBI rats had longer cell body perimeters than shams at 1D (95% CI: 2–23, *P* > 0.99, *K* > 100), 7D (95% CI: −1 to 15, *P* = 0.98, *K* = 61.83), and 43D post-injury (95% CI: −6 to 17, *P* = 0.92, *K* = 11.75), respectively (*d* = 0.02–0.03; Figure 3J). Additionally, TBI rats at 1D had longer cell body perimeters than TBI rats at 2H, 7D, and 25D post-injury (95% CI range: 2–22, *P* > 0.99, *K* > 100; Figure 3J). Similarly, TBI rats had larger cell body areas than shams at 1D (95% CI: −4 to 88, *P* > 0.99, *K* > 100), 7D (95% CI: −17 to 62, *P* = 0.96, *K* = 22.53), and 43D post-injury (95% CI: −34 to 83, *P* = 0.91, *K* = 10.02), respectively (*d* < 0.01; Figure 3M). Further, TBI rats at 1D had larger cell body areas than TBI rats at 2H, 7D, and 25D post-injury (95% CI range: −12 to 78, *P* = 0.98, *K* = 52.10; Figure 3M).

PND17 TBI rats had longer cell body perimeters than PND17 shams (95% CI: −3 to 11, *P* = 0.93, *K* = 12.26) and PND35 TBI rats had longer cell body perimeters than PND35 shams (95% CI: 2–18, *P* > 0.99, *K* > 100), respectively (*d* = 0.01–0.02; Figure 3K). Additionally, PND35 TBI rats had longer cell body perimeters than PND17 TBI rats (95% CI: −3 to 13, *P* = 0.96, *K* = 24.53, *d* = 0.01; Figure 3K). In contrast, there were no differences in cell body area between TBI and sham rats at PND17 (*P* = 0.83, *K* = 2.56) or between PND17 TBI rats PND35 TBI rats (*P* = 0.88, *K* = 4.97; Figure 3N). However, PND35 TBI rats had a larger cell body area than PND35 shams (95% CI: −13 to 68, *P* = 0.98, *K* = 70.43, *d* = 0.003; Figure 3N).

TBI rats had longer cell body perimeters than shams at terminal ages PND35 (95% CI: 1–23, *P* > 0.99, *K* > 100) and PND42 (95% CI: −3 to 16, *P* = 0.95, *K* =15.42), respectively (*d* = 0.02–0.03; Figure 3L), and there were no other perimeter differences between TBI and sham rats. Additionally, TBI rats at terminal age PND24 had shorter cell body perimeters than TBI rats at all other terminal ages (95% CI range: 4–22, *P* > 0.99, *K* > 100, *d* ≤ 0.04; Figure 3L). There were no differences in cell body area between TBI and shams across all terminal ages (*P* < 0.90, *K* ≤ 3.00; Figure 3O); however, TBI rats at PND24 had smaller cell body areas than TBI rats at PND35, PND42, and PND60, but not PND17 (95% CI range: 7–95, *P* > 0.99, *K* > 100; Figure 3O).

### Diffuse TBI Activated Microglia in the S1BF of Rats Injured at Both PND17 and PND35

**Figure 4:**
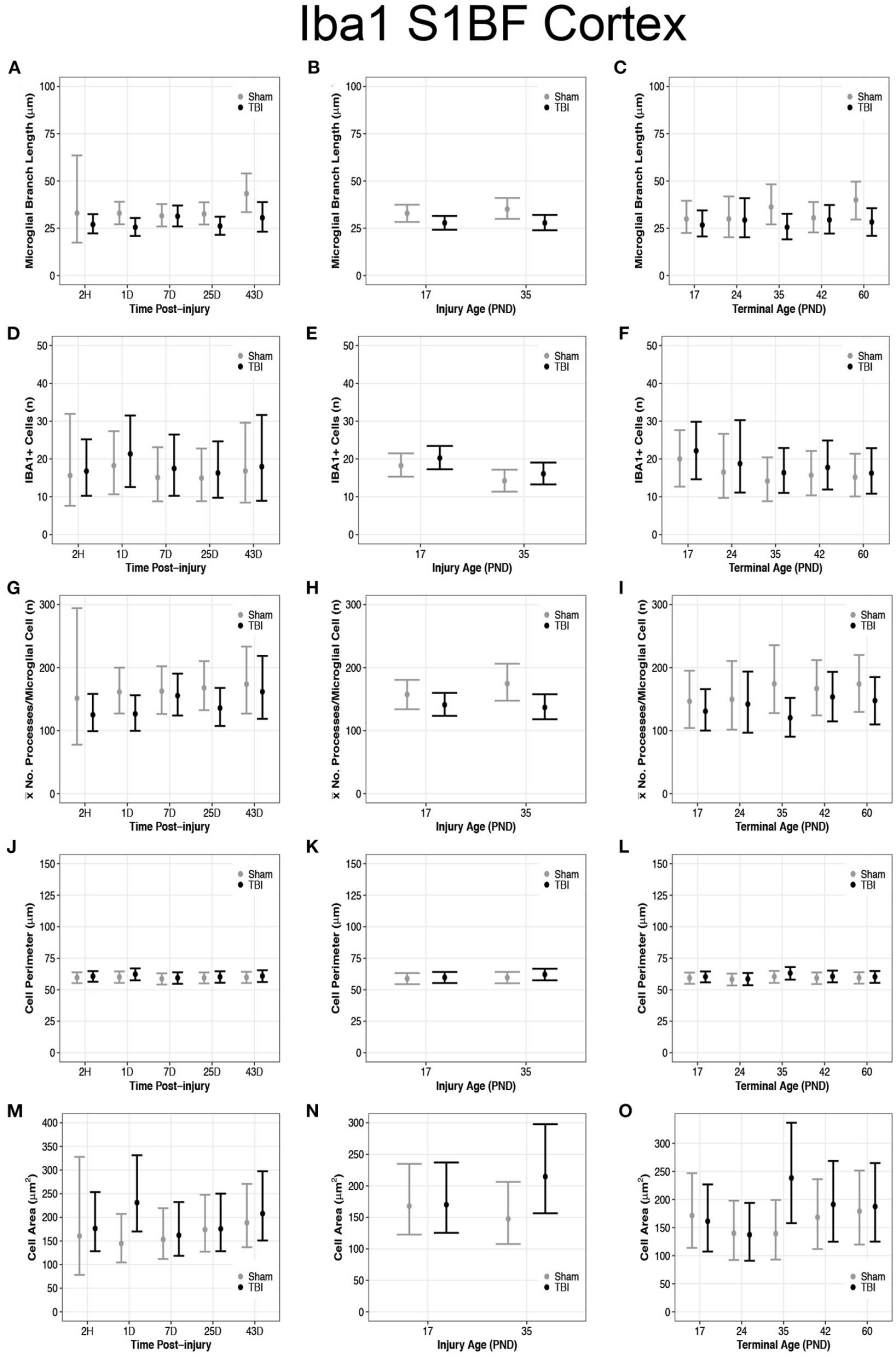
Diffuse TBI activated microglia in the S1BF of rats injured at PND17 and PND35. Point estimates (posterior means) and corresponding 95% credible intervals (highest posterior density intervals) are presented. **(A)** TBI rats at 1D, 25D, and 43D post-injury had shorter microglial branch lengths than shams. **(B)** TBI rats had shorter microglial branch lengths than shams at both injury ages PND17 and PND35. **(C)** TBI rats at terminal ages PND35 and PND60 had shorter microglial branch lengths than shams. **(D–F)** There were no differences in cell number between sham and TBI across times post-injury, injury ages, or terminal ages. **(E)** Both sham and TBI rats had fewer cells at injury age PND35 compared to PND17. **(G)** TBI rats had fewer mean processes per microglial cell than shams at 1D and 25D post-injury. **(H)** TBI rats had fewer mean processes per microglia cell than shams at both injury age PND17 and PND35. **(I)** TBI rats at terminal age PND35 had fewer mean processes per cell than shams. **(J–L)** TBI did not affect cell perimeter. **(M)** TBI rats at 1D had a larger cell area than sham. **(N)** PND35 TBI rats had a larger cell area than PND35 shams and PND17 TBI. **(O)** TBI rats at terminal age PND35 had a larger cell area than shams, and TBI rats at PND60 had a larger area than TBI rats at PND17.

Overall, there were more microglia in the S1BF of PND17 rats compared to the S1BF of PND35 rats, suggesting a greater inflammatory response in PND17 rats. TBI rats at 1D (95% CI: 1–13, *P* > 0.99, *K* > 100), 25D (95% CI: 0–13, *P* = 0.99, *K* > 100), and 43D post-injury (95% CI: 2–23, *P* > 0.99, *K* > 100) had shorter microglial branch lengths than shams, respectively (*d* = 0.13–0.18; Figure 4A). PND17 TBI rats had shorter microglia branch lengths in the S1BF than PND17 shams (95% CI: 0–10, *P* > 0.99, *K* > 100) and PND35 TBI rats had shorter branch lengths than PND35 shams (95% CI: 1–13, *P* > 0.99, *K* > 100), respectively (*d* = 0.10; Figure 4B). TBI rats at terminal ages PND35 (95% CI: 0–21, *P* > 0.99, *K* > 100) and PND60 (95% CI: 2–21, *P* > 0.99, *K* > 100) had shorter microglial branch lengths than shams, respectively (*d* = 0.11–0.14; Figure 4C).

There were no differences in cell number between sham and TBI across times post-injury, injury ages, or terminal ages (Figures 4D–F). However, both PND35 TBI and PND35 shams had fewer cells (95% CI: 0–8, *P* = 0.97, *K* = 30.50) than at PND17 (95% CI: 0–8, *P* = 0.98, *K* = 41.18), respectively (*d* = 0.10–0.13; Figure 4E).

TBI rats had fewer mean processes per microglial cell than shams at 1D (95% CI: −5 to 75, *P* = 0.98, *K* = 49.5) and 25D post-injury (95% CI: −11 to 75, *P* = 0.97, *K* = 29.61, *d* = 0.02; Figure 4G). PND17 TBI rats had fewer mean processes per microglia cell than PND17 shams (95% CI: −11 to 44, *P* = 0.92, *K* = 11.40), and PND35 TBI rats had fewer mean processes than PND35 shams (95% CI: 5–71, *P* > 0.99, *K* > 100), respectively (*d* = 0.01–0.02; Figure 4H). TBI rats at terminal age PND35 had fewer mean processes per microglial cell than shams (95% CI: 1–107, *P* = 0.99, *K* > 100, *d* = 0.03; Figure 4I). Rod microglia, a microglial phenotype associated with pathology (59, 60), were observed in the S1BF of PND17 rats but not in PND35 rats (Figure 2B). Although rod microglia were not observed in cortical regions of interest in PND35 rats, this is not evidence for their absence in PND35 rats and therefore full brain analysis is warranted to reach that conclusion.

### Diffuse TBI Increased Microglial Cell Area but Not Cell Perimeter Size in the S1BF of Rats Injured at Both PND17 and PND35

Overall, microglia in the S1BF of PND35 rats had an increased cell body area compared to the S1BF of PND17 rats, suggesting a greater phagocytic potential in PND35 rats. TBI did not affect cell perimeter in the S1BF (Figures 4J–L), however, our data support TBI increased cell area in the S1BF (Figures 4M–O). There were no differences in cell body perimeter length between TBI and sham rats across times post-injury (Figure 4J); however, TBI rats at 1D post-injury had larger cell body areas than shams (95% CI: 28–145, *P* > 0.99, *K* > 100; *d* = 0.01; Figure 4M). There were no differences in cell body perimeter lengths between TBI and sham rats, or between TBI rats, at injury ages PND17 or PND35 (*P* < 0.90, *K* ≤ 3.00; Figure 4K). However, PND35 TBI rats had larger cell body areas than both PND35 and PND17 shams (95% CI: 19–115, *P* > 0.99, *K* > 100), and PND17 TBI rats (95% CI: −3 to 92, *P* > 0.99, *K* > 100, *d* < 0.01; Figure 4N).

There were no differences in cell body perimeter length between TBI and sham rats across terminal ages (Figure 4L), but TBI rats at terminal age PND35 had larger cell body areas than shams (95% CI: 42–156, *P* > 0.99, *K* > 100, *d* = 0.01; Figure 4O). Additionally, cell body area increased in TBI rats across time, given TBI rats at terminal age PND60 had larger cell body areas than TBI rats at terminal age PND17 (95% CI: −20 to 72, *P* = 0.95, *K* = 16.97, *d* < 0.01; Figure 4O).

### Diffuse TBI Activated Microglia in the Perirhinal Cortex of Rats Injured at Both PND17 and PND35

**Figure 5:**
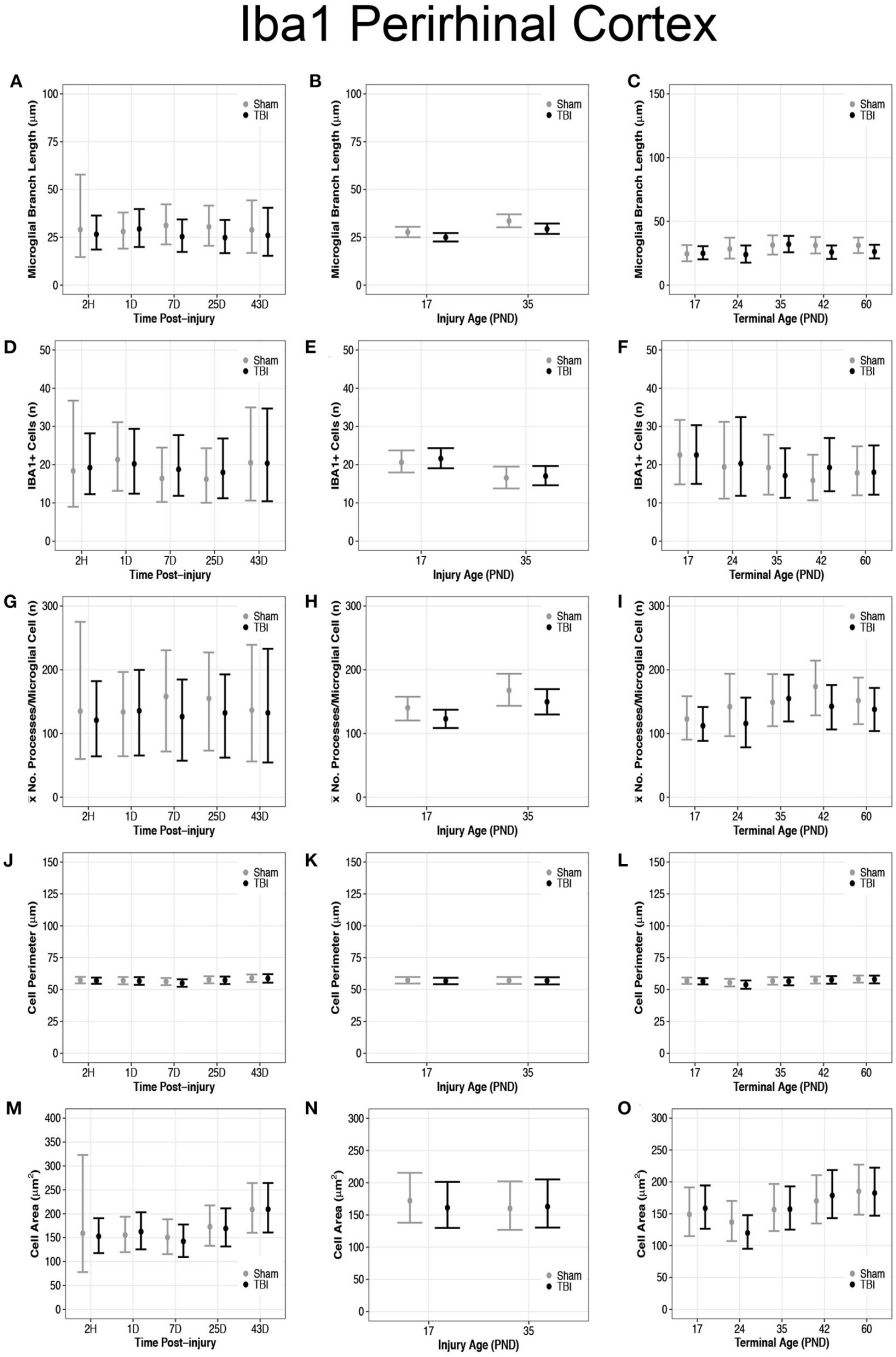
Diffuse TBI activated microglia in the perirhinal cortex of rats injured at PND17 and PND35. Point estimates (posterior means) and corresponding 95% credible intervals (highest posterior density intervals) are presented. **(A)** There were no differences in microglial branch lengths between sham and TBI across times post-injury. **(B)** TBI rats had shorter microglial branch lengths compared to shams at injury ages PND17 and PND35. PND35 TBI rats had longer microglial branch lengths than PND17 TBI rats. **(C)** TBI rats had shorter microglial branch lengths than shams at terminal ages PND24, PND42, and PND60. **(D–F)** There were no differences in the number of microglia between sham and TBI rats across times post-injury, injury ages, and terminal ages. **(E)** Both sham and TBI rats at PND35 had fewer microglia than PND17 rats. **(G)** The mean number of processes per microglia in sham and TBI rats across times post-injury were similar. **(H)** TBI rats had fewer mean processes per microglia than shams at injury ages PND17 an PND35, and PND35 TBI rats had more mean processes per microglia than PND17 TBI rats. **(I)** TBI rats had fewer mean processes per microglia than shams at terminal ages PND24 and PND42. **(J–L)** There were no differences between TBI and sham rats in cell body perimeter lengths among times post-injury, injury ages, or terminal ages. **(M)** Both TBI and sham rats at 43D post-injury had larger cell body areas than other times post-injury. **(N)** There were no differences in cell body area between TBI and sham rats between injury ages. **(O)** TBI rats at PND24 had smaller cell body area than other time points, and both TBI and sham rats at PND60 had larger cell body areas than PND17, PND24, and PND35.

Overall, there were more microglia in the perirhinal cortex of PND17 rats that had shorter branches and fewer processes compared to PND35, suggesting that TBI caused de-ramification and microglial activation in PND17 rats compared to PND35 rats in the perirhinal cortex. There were no differences in microglial branch lengths between sham and TBI across times post-injury (Figure 5A). PND17 TBI rats had shorter microglial branch lengths than PND17 shams (95% CI: −1 to 6, *P* = 0.95, *K* = 20.43) and PND35 TBI rats had shorter branch lengths than PND35 shams (95% CI: 0–9, *P* = 0.98, *K* = 52.81), respectively **(***d* = 0.10–0.11; Figure 5B). Additionally, PND35 TBI rats had longer microglial branch lengths than PND17 TBI rats (95% CI: 1–8, *P* = 0.99, *K* > 100, *d* = 0.13; Figure 5B). TBI rats had shorter microglial branch lengths than shams at terminal ages PND24 (95% CI: −4 to 13, *P* = 0.91, *K* = 10.40), PND42 (95% CI: 95% CI: −1 to 12, *P* = 0.98, *K* = 60.86) and PND60 (95% CI: −2 to 12, *P* = 0.98, *K* = 49.85), respectively (*d* = 0.09–0.10; Figure 5C).

There were no differences in number of microglia between sham and TBI rats across times post-injury, injury ages, and terminal ages (Figures 5D–F). However, PND35 shams (95% CI: 0–8, *P* = 0.98, *K* = 47.39) and PND35 TBI rats (95% CI: 1–8, *P* = 0.99, *K* = 92.75) had fewer microglia, respectively, compared to PND17 (*d* = 0.14; Figure 5E).

There were no differences in mean number of processes per microglial cell between sham and TBI rats across times post-injury (Figure 5G). TBI rats had fewer mean processes per microglial cell than shams at injury ages PND17 (95% CI: −4 to 38, *P* = 0.98, *K* = 52.57) and PND35 (95% CI: −10 to 46, *P* = 0.95, *K* = 20.78), respectively (*d* = 0.01–0.02; Figure 5H). Additionally, PND35 TBI rats had more mean processes per microglial cell than PND17 TBI rats (95% CI: 5–48, *P* = 0.98, *K* = 61.50, *d* = 0.02; Figure 5H). TBI rats had fewer mean processes per microglial cell than shams at terminal ages PND24 (95% CI: −22 to 75, *P* = 0.96, *K* = 23.05) and PND42 (95% CI: −11 to 73, *P* = 0.99, *K* = 84.11, *d* = 0.02; Figure 5I).

### Diffuse TBI Did Not Affect Microglial Cell Perimeter or Cell Area Size in the Perirhinal Cortex of Rats Injured PND17 or PND35

There were no differences in cell body perimeter lengths among times post-injury, injury ages, or terminal ages in the perirhinal cortex (Figures 5J–L). Both TBI (95% CI: 3–91, *P* > 0.99, *K* > 100) and sham rats (95% CI: 9–98, *P* > 0.99, *K* > 100) at 43D post-injury had larger cell body areas than other times post-injury (*d* = 0.01; Figure 5M), which suggests increasing cell body size with time; however, there were no injury-induced differences in area between injury ages (Figure 5N).

There were no differences in cell body area between TBI and sham rats at each terminal age (*P* ≤ 0.91, *K* ≤ 4.00), but TBI rats at PND24 had a smaller cell body area than other time points (95% CI: 8–69, *P* > 0.99, *K* > 100), and both TBI and sham rats at PND60 had larger cell body areas than PND17, PND24, and PND35 (95% CI: −10 to 61, *P* = 0.98, *K* = 45.33, *d* = 0.006–0.01; Figure 5O). These findings further support increasing cell body size with time.

### Diffuse TBI Did Not Affect GFAP Expression in the Peri-Injury Cortex of Rats Injured at PND17 or PND35

**Figure 6:**
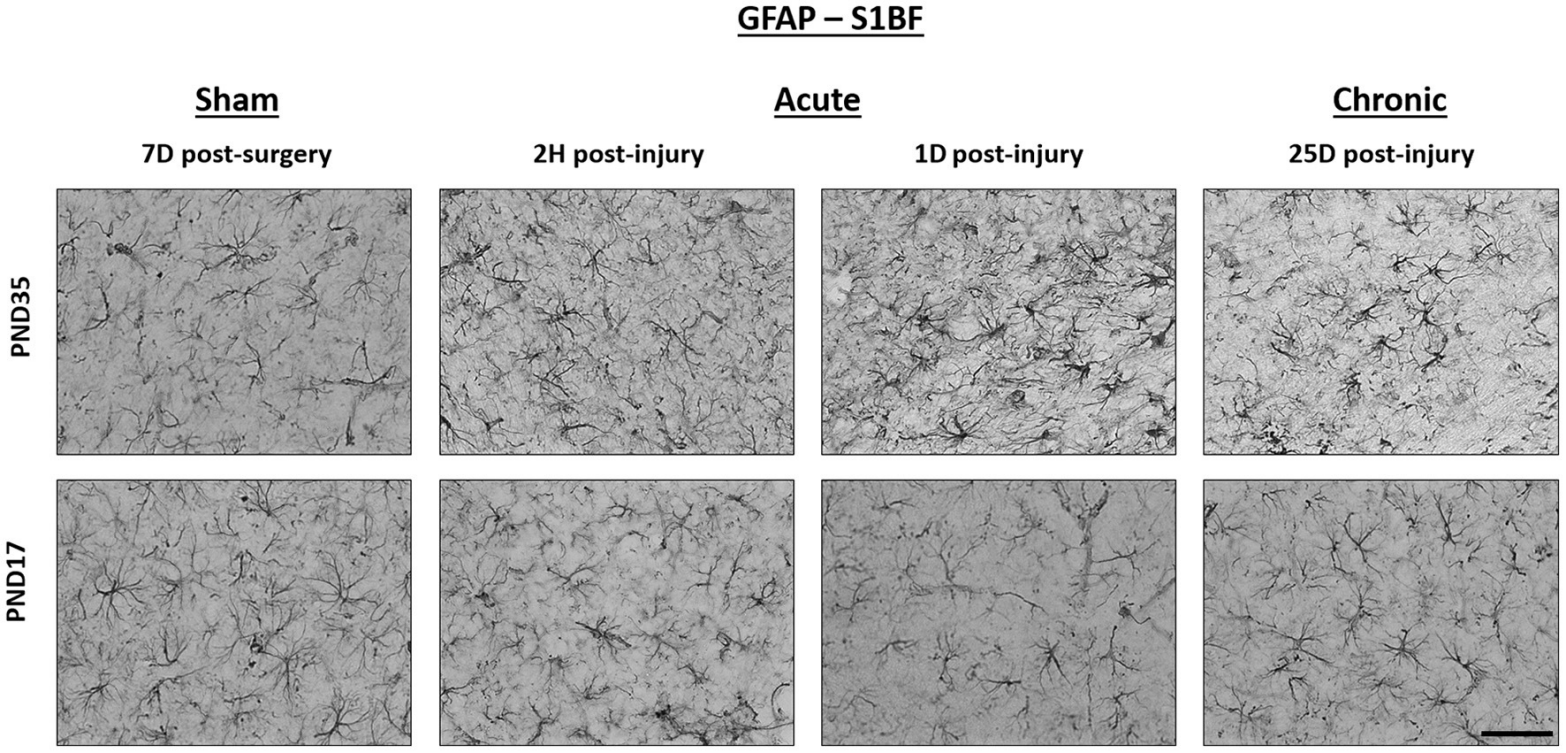
TBI caused no detectable difference in GFAP expression in PND17 or PND35 rats. Immunohistochemistry was used to examine GFAP expression. Representative images shown are from both PND17 and PND35 injured rats at 7 days post-sham surgery and 2 hours (H), 1 day (D), and 25D post-injury. All representative images are from the S1BF. Scale bar = 50 μm. (sham *n* = 39, TBI *n* = 35).

**Figure 7:**
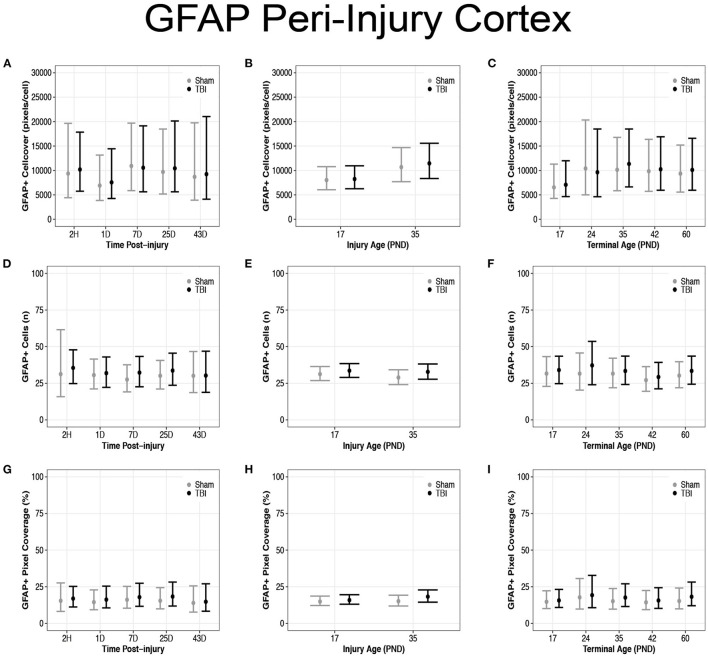
Diffuse TBI did not affect GFAP expression in the peri-injury cortex. Point estimates (posterior means) and corresponding 95% credible intervals (highest posterior density intervals) are presented. There were no differences between sham and TBI rats across times post-injury, injury ages, or terminal ages **(A–C)** in cell coverage, **(D–F)** GFAP+ cell number, **(G–I)** or pixel coverage values.

There were no differences in cell coverage between sham and TBI across time post-injury, injury ages, or terminal ages (Figures 6, 7A–C). There were also no differences in number of GFAP + cells between sham and TBI across times post-injury, injury ages, and terminal ages (Figures 7D–F). Sham and TBI also had similar pixel coverage values across time post-injury, injury ages, and terminal ages (Figures 7G–I).

### Diffuse TBI Did Not Affect GFAP Expression in the S1BF of Rats Injured at PND17 or PND35

**Figure 8:**
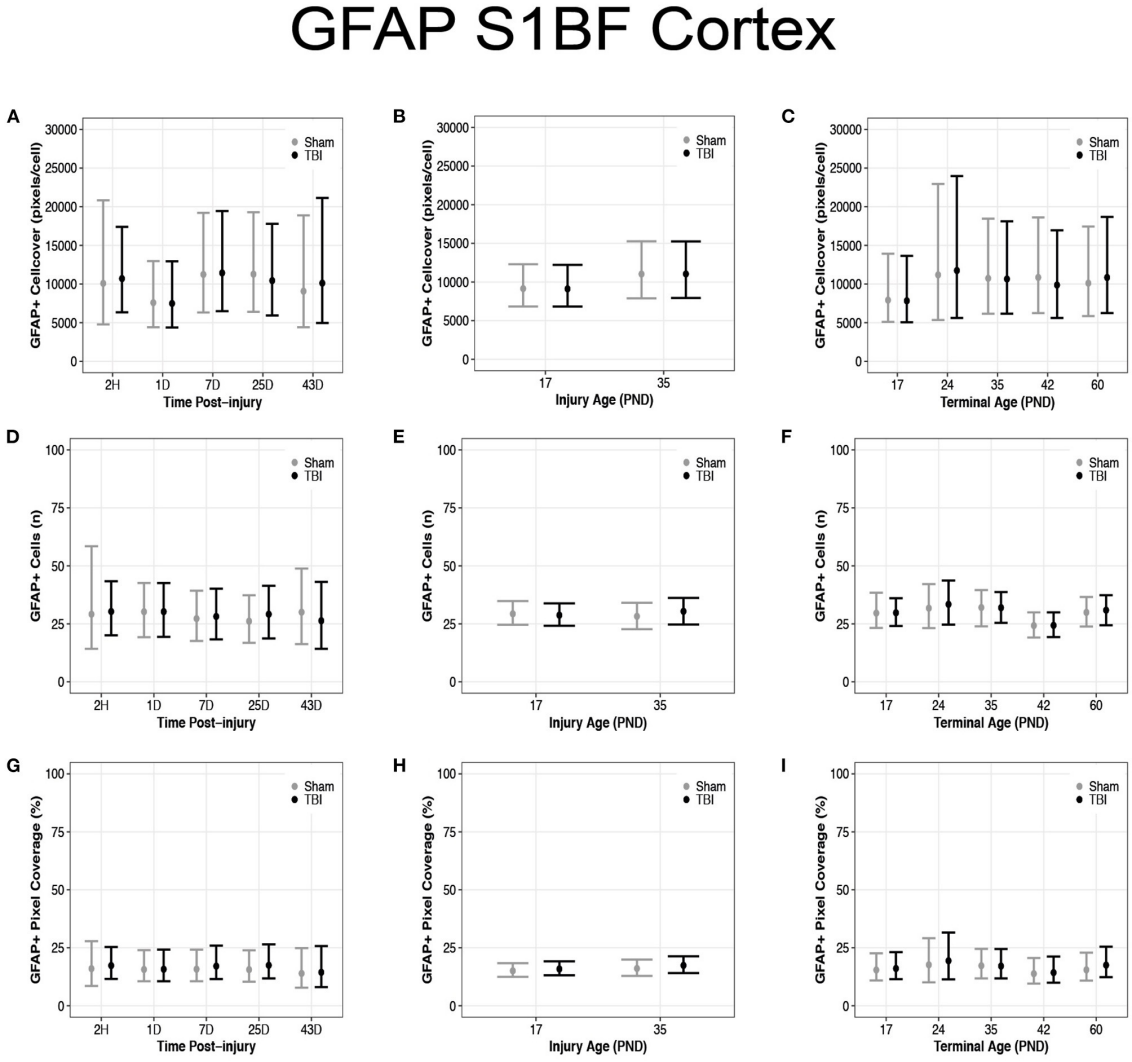
Diffuse TBI did not affect GFAP expression in the S1BF. Point estimates (posterior means) and corresponding 95% credible intervals (highest posterior density intervals) are presented. **(A–C)** There were no differences in cell coverage between sham and TBI rats across times post-injury, injury ages, and terminal ages. **(D,E)** There were no differences in the number of GFAP + cells between sham and TBI rats across times post-injury or injury ages, **(F)** however, at terminal age PND42, both sham and TBI rats had fewer GFAP + cells than other terminal ages. **(G–I)** Sham and TBI rats had similar pixel coverage values across terminal ages, injury ages, and times post-injury.

There were no differences in cell coverage between sham and TBI across times post-injury, injury ages, and terminal ages in the S1BF (Figures 8A–C). There were also no differences in number of GFAP + cells between sham and TBI across times post-injury, injury ages and terminal ages (Figures 8D–F). However, both sham (95% CI: −1 to 16, *P* = 0.95, *K* = 18.45) and TBI rats (95% CI: 0–15, *P* = 0.97, *K* = 29.15) had fewer GFAP + cells at terminal age PND42 (*d* = 0.13–0.16; Figure 8F). Sham and TBI also had similar pixel coverage values across terminal ages, injury ages, and times post-injury (Figures 8G–I).

### Diffuse TBI Did Not Affect GFAP Expression in the Perirhinal Cortex of Rats Injured at PND17 or PND35

**Figure 9:**
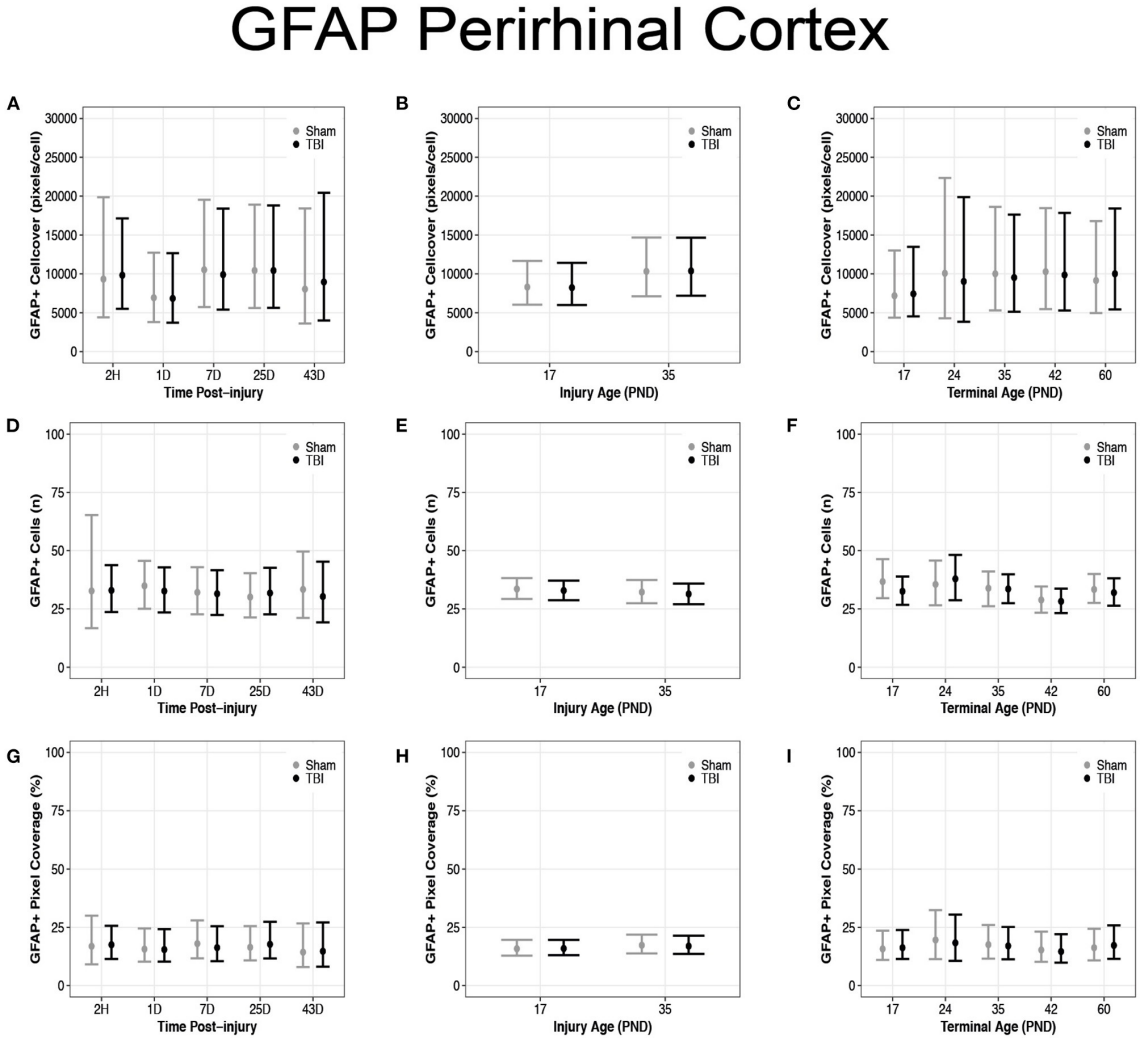
Diffuse TBI did not affect GFAP expression in the perirhinal cortex. Point estimates (posterior means) and corresponding 95% credible intervals (highest posterior density intervals) are presented. There were no differences between sham and TBI rats across time post-injury, injury ages, or terminal ages **(A–C)** in cell coverage, **(D–F)** GFAP + cell number, **(G–I)** or pixel coverage values.

There were no differences in GFAP + cell coverage between sham and TBI rats across time post-injury, injury ages, and terminal ages in the perirhinal cortex (Figures 9A–C). There were also no differences in number of GFAP + cells between sham and TBI rats across time post-injury, injury ages, or terminal ages (Figures 9D–F). Sham and TBI rats also had similar pixel coverage values across time post-injury, injury ages, and terminal ages (Figures 9G–I).

## Discussion

**Figure 10:**
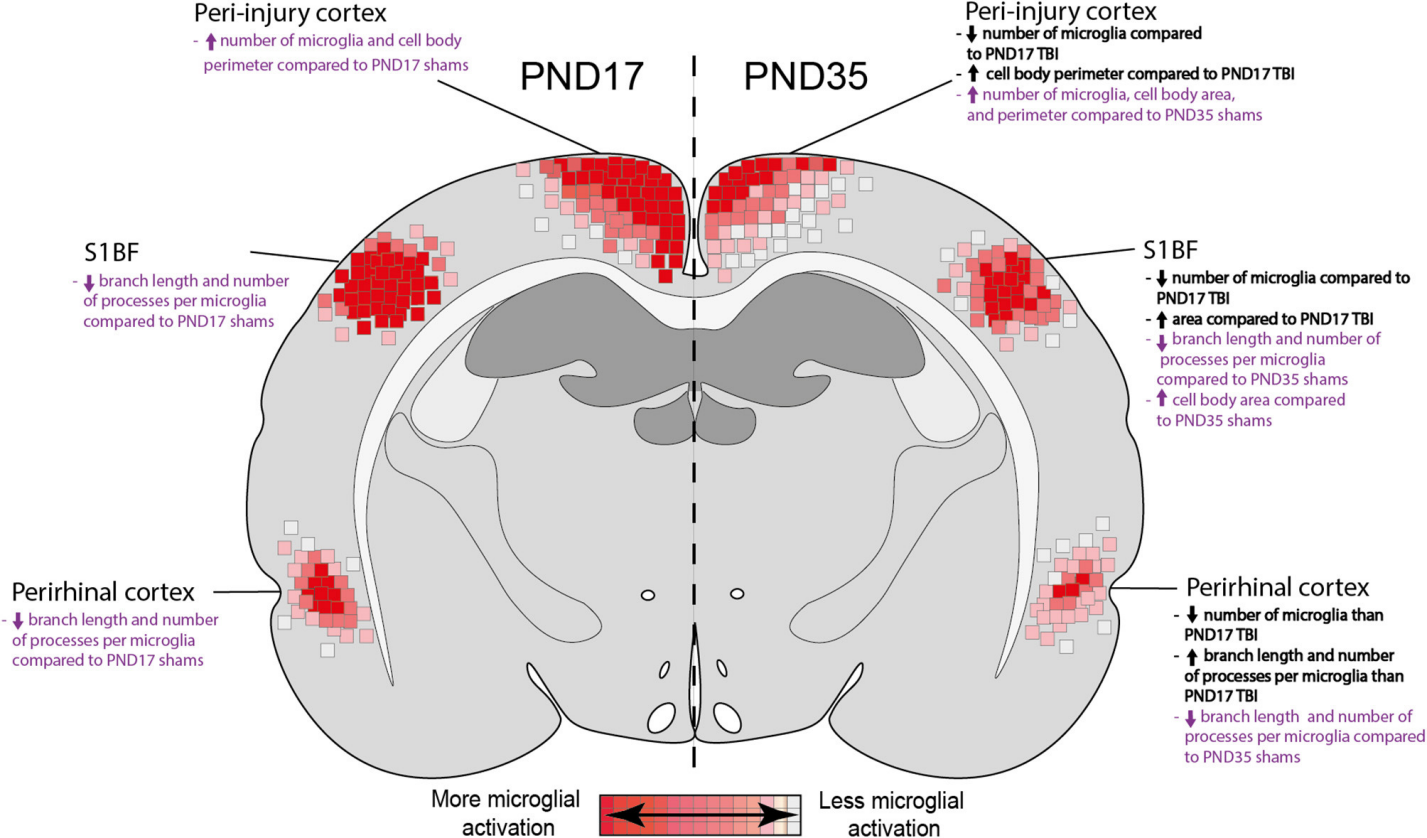
Summary heat map of microglial activation in PND17 rats compared to PND35. Quantitative skeletal analysis was used to assess microglia morphology (number of microglia, number of microglia branches, branch processes per cell) and results are summarized in a heat map. Results in bold black text indicate differences between PND35 TBI and PND17 TBI. Results in purple text indicate differences between TBI groups and their respective shams. Overall, TBI activated microglia in both ages. Rats injured at PND35 had fewer microglia in all cortical regions of interest compared to rats injured at PND17. PND17 had more microglial activation (de-ramification) compared to rats injured at PND35, indicated by at least one measured outcome in each cortical region of interest.

Microglia play a key role in both the positive and negative effects of inflammation post-injury (61). It is important to understand microglial physiology under non-inflammatory and inflammatory conditions during early life. In this study, we found differences in the microglial response between PND17 and PND35 rats (both injured and sham) throughout the cortex using microglial branch length, microglial processes per cell, and microglial abundance as quantitative outcomes (Figure 10). Our results show that TBI activated microglia in both PND17 and PND35 rats in comparison to uninjured shams. To our knowledge, this is the first study to comprehensively examine microglial morphology after mFPI in PND17 and PND35 rats at acute and sub-acute time points.

In agreement with previously published studies, we detected a change in microglial morphology and/or abundance after TBI regardless of age-at-injury (13, 28, 62). Similar to recent clinical observations (63), we found that microglial activation quickly ensued after TBI (1D post-injury) and was maintained into early adulthood (60D post-injury). This chronic maintenance of an activated morphology is supported by both pre-clinical and clinical data, as activated microglia have been observed in patients 17-years post-injury, and we have reported microglia activation at 9 months post-injury in rats (13, 64, 65). Such prolonged microglial activation is often associated with negative developmental outcomes, such as chronic behavioral deficits, progressive cortical thickness reduction, decreased corpus callosum area, neurodegeneration, and cognitive deficits (24, 66–69). In the clinic, behavioral and affective outcomes are exacerbated in patients injured at a younger age; therefore, our study introduces a plausible, microglia-centric reason for this (3, 66, 70).

In all regions tested, our results show that there were more microglial cell bodies in PND17 rats compared to PND35 rats, independent of injury. This suggests that, regardless of injury status, PND17 rats may have a greater density of microglia. The increased number of microglia may be the result of overshoot in microglia observed in early post-natal life (71). Alternatively, the number of microglia may decrease after they fulfill their role in neuronal circuit formation in the developing brain (postnatal weeks 1–3 in the rodent) (72, 73). We also found more microglial activation in the perirhinal cortex in PND17 rats compared to PND35 rats, which may be due to the increased circuit remodeling during this developmental period (74), as microglia have been shown to reach their full ramification level by PND14 (75). Furthermore, we observed the presence of rod microglia in the S1BF of PND17 rats but not PND35 rats. The rod microglial morphology is associated with inflammation and pathology; however, their exact role after TBI is unknown (59, 60, 76). The presence of rod microglia in PND17 brain-injured rats, but not PND35 rats, supports a differential immune response to diffuse TBI dependent on age-at-injury, where young rats may be more vulnerable to inflammatory pathology.

We examined the microglia cell body size (area and perimeter) because a larger cell body is a hallmark feature of microglia activation (77). Overall, TBI rats had a larger cell body (measured by area and perimeter) than sham rats, indicating that microglia adopt and sustain an activated phenotype after experimental TBI (sustained until PND60 in the peri-injury cortex). Post-injury enlargement of microglia cell soma size and ameboid morphology have been well-documented (78, 79). This is likely because of increased phagocytic activity of microglia in pathological conditions (15), though the size is rarely quantified. Rats subjected to TBI had larger cell bodies than shams in the peri-injury and S1BF cortices, and the cells were largest at 1D post-injury. This suggests that microglia may respond quickly to the excitotoxicity, cell death, and off-set homeostasis associated with TBI (80).

Although the primary objective of this study was to investigate the effects of age-at-injury on glial activation after diffuse TBI, we utilized a unique study design that also allowed evaluation of glial activation as a function of both time post-injury and animal age at the time of tissue collection (terminal age). In both the peri-injury cortex and the S1BF, there was extensive microglial activation at 1, 7, and 25 DPI that was, in part, resolved by 43 DPI. Minimal support existed for terminal age influencing microglial activation. Overall, we conclude that time post-injury had a greater impact on the microglial response to diffuse TBI compared to terminal age. Microglia were activated immediately after injury, with peak activation at 1 DPI. This result is congruous with our previous studies that showed microglial activation and number increase immediately after TBI and peak within the first 7 days post-injury (81, 82).

We also investigated whether age influenced microglia cell size, and if microglia grew as a rat aged. Our data did not support our hypothesis that microglia cell somas increase in size with brain growth. Some of our results weakly suggested that microglial cell bodies grew with time and were limited in the amount that their cell soma could grow during microglial activation at younger ages. For example, we found that in the peri-injury and S1BF cortices, microglial cell body perimeter and area were larger in PND35 rats than in PND17 rats. Overall, our evidence suggests that microglial cell bodies do not significantly grow between PND17 and PND60. These findings support the work of Dos Santos et al., who determined that microglial cell size is governed by a mechanism that evolved >200 million years ago and is not dependent on brain size (83). Microglial cell body size throughout post-natal development has not been well-documented and, to our knowledge, this is the first study to detail this in a TBI model. However, we caution that the effect sizes for our data were small, so any conclusions about cell soma size warrant further investigation.

Astrocytes become hypertrophic in response to injury (84). Astrocytes contain an intracellular network of the intermediate fiber GFAP, and upon mechanical stress, GFAP expression is upregulated (85–87); hence, GFAP has been heavily investigated as a blood biomarker of TBI (88, 89). We found no differences in GFAP expression between sham and TBI rats across time post-injury, terminal age, or injury age. These results are similar to our previously reported data where we observed no injury-induced changes in GFAP immunoreactivity at chronic time points compared to uninjured rats (13). Although GFAP upregulation is a common marker for distressed astrocytes (85, 86), it does not assess for astrocyte morphology. Considering there are up to eight distinct astrocyte morphologies (90), it is possible that the morphology changes in response to TBI without a detectable change in GFAP expression. Therefore, although our results suggest a negligible astrocytic response to TBI in these age groups, we cannot rule out morphological changes.

A primary limitation of this study was the use of only male rats, considering there is evidence of sex differences in cellular and systemic outcomes of TBI (37). Our ongoing studies in juvenile rats include sex as a biological variable that affects behavior and neuropathology. In the current study, we focused on cortical regions, but we have previously reported glial changes in white matter (13). Additional studies are needed to investigate the acute and sub-acute glial response in white matter tracts, as they likely differ substantially from those in gray matter in terms of both magnitude and phenotype. Another limitation of the current study is that the glial response to TBI was examined as an isolated event and future studies that investigate how glial activation influences neuropathology and functional outcome are warranted. We have previously shown that brain injury at PND17 or PND35 resulted in acute and sub-acute cognitive, motor, and affective deficits compared to adult-injured and naïve rats (24). We have also shown that regardless of age-at-injury, diffuse TBI resulted in neuropathology in juvenile rats (13). Based on those previous studies, glial activation likely contributes to TBI-induced behavioral deficits and neuropathology.

As with any histological study, there is the added limitation of working with 2-dimensional images in a 3-dimensional space. This means that cells could be sliced in different planes, rather than centrally, thereby increasing the probability of larger cells being caught in the slice. To combat this, and to ensure our images accurately represented the biological specimen, we used Z-stacked images that spanned the 40 μm tissue slice [microglial are around 15–30 μm in width/diameter (91), with the exception of rod microglia which have an elongated cell body (76)], and adhered to the rules of Nyquist sampling. Furthermore, despite the moderate to strong support for multiple differences between groups in this study, many effect sizes were small, which indicates that additional data need to be collected to further evaluate the magnitude and importance of identified effects.

## Conclusions and Future Directions

In conclusion, we found evidence that rats injured at PND17 had increased widespread microglial activation. A more activated phenotype was also seen in shams at PND17, but injury still further decreased the amount of ramification. This suggests some physiological differences between microglia in the PND17 rat compared to microglia in the PND35 rat, and that injury might cause more de-ramification in PND17 microglia. We therefore conclude that age-at-injury significantly influenced the microglial response to TBI. Such de-ramification of younger microglia and their smaller cell body size may render them less able to respond to injury and therefore make them more vulnerable to injury-induced physiological and affective deficits.

## Author's Note

RR was an employee of Phoenix Children's Hospital during the data collection for this study.

## Data Availability Statement

The datasets presented in this study can be found in online repositories. The name of the repository and accession number can be found below: Dryad, https://doi.org/10.5061/dryad.5tb2rbp4r.

## Ethics Statement

The animal study was reviewed and approved by Institutional Animal Care and Use Committee (IACUC) at the University of Arizona (protocol 13–460). The Animal Research: Reporting *in vivo* Experiments (ARRIVE) guidelines were followed.

## Author Contributions

TG helped execute the experiments, led data collection, analyzed data, constructed figures, and led manuscript writing. SM analyzed data, helped construct figures, and assisted with writing and editing the manuscript. JO helped collect data and assisted with writing and editing the manuscript. RR conceived and designed the study, executed the experiments, analyzed data, constructed figures, and assisted with writing and editing the manuscript. All authors contributed to the article and approved the submitted version.

## Funding

This study was supported by a grant from the National Institute of Neurological Disorders and Stroke (R21NS120022 to RR) and the University of Arizona College of Medicine—Phoenix and Phoenix Children's Hospital Mission Support (RR).

## Conflict of Interest

The authors declare that the research was conducted in the absence of any commercial or financial relationships that could be construed as a potential conflict of interest.

## Publisher's Note

All claims expressed in this article are solely those of the authors and do not necessarily represent those of their affiliated organizations, or those of the publisher, the editors and the reviewers. Any product that may be evaluated in this article, or claim that may be made by its manufacturer, is not guaranteed or endorsed by the publisher.

## Supplementary Material

Supplementary Figure 1 Iba1 stained microglia in the peri-injury cortex at 2 h (H), 1 day (D), and 25D post-injury compared to uninjured shams at 7D. All representative images were taken in the peri-injury cortex. Scale bars = 50 μm (sham *n* = 39, TBI *n* = 35).

Supplementary Figure 2 Iba1 stained microglia in the perirhinal cortex at 2 h (H), 1 day (D), and 25D post-injury compared to uninjured shams at 7D. All representative images were taken in the perirhinal cortex. Scale bars = 50 μm (sham *n* = 39, TBI *n* = 35).

Supplementary Figure 3 GFAP stained microglia in the peri-injury cortex at 2 h (H), 1 day (D), and 25D post-injury compared to uninjured shams at 7D. All representative images were taken in the peri-injury cortex. Scale bars = 50 μm (sham *n* = 39, TBI *n* = 35).

Supplementary Figure 4 GFAP stained microglia perirhinal cortex at 2 h (H), 1 day (D), and 25D post-injury compared to uninjured shams at 7D. All representative images were taken in the perirhinal cortex. Scale bars = 50 μm (sham *n* = 39, TBI *n* = 35).

## Abbreviations

ABC: Avidin-biotin complex
ARRIVE: Animal Research: Reporting *in vivo* Experiments
D: Day
DAB, 3: 3′-diam-inobenzidine
FPI: Fluid percussion injury
GFAP: Glial fibrillary acidic protein
H: Hour
IACUC: Institutional Animal Care and Use Committee
Iba1: Ionized calcium binding adaptor molecule 1
mFPI: Midline fluid percussion
NHS: Normal horse serum
PBS: Phosphate buffered saline
PFA: Paraformaldehyde
pH: Potential hydrogen
PND: Post-natal day
S1BF: Primary somatosensory barrel field cortex
TBI: Traumatic brain injury.
