# Supplementary figures and images for: Age-At-Injury Influences the Glial Response to Traumatic Brain Injury in the Cortex of Male Juvenile Rats
Source: Front Neurol. 2022 Jan 17;12:804139. doi: 10.3389/fneur.2021.804139 (PMC8802670; doi:10.3389/fneur.2021.804139)

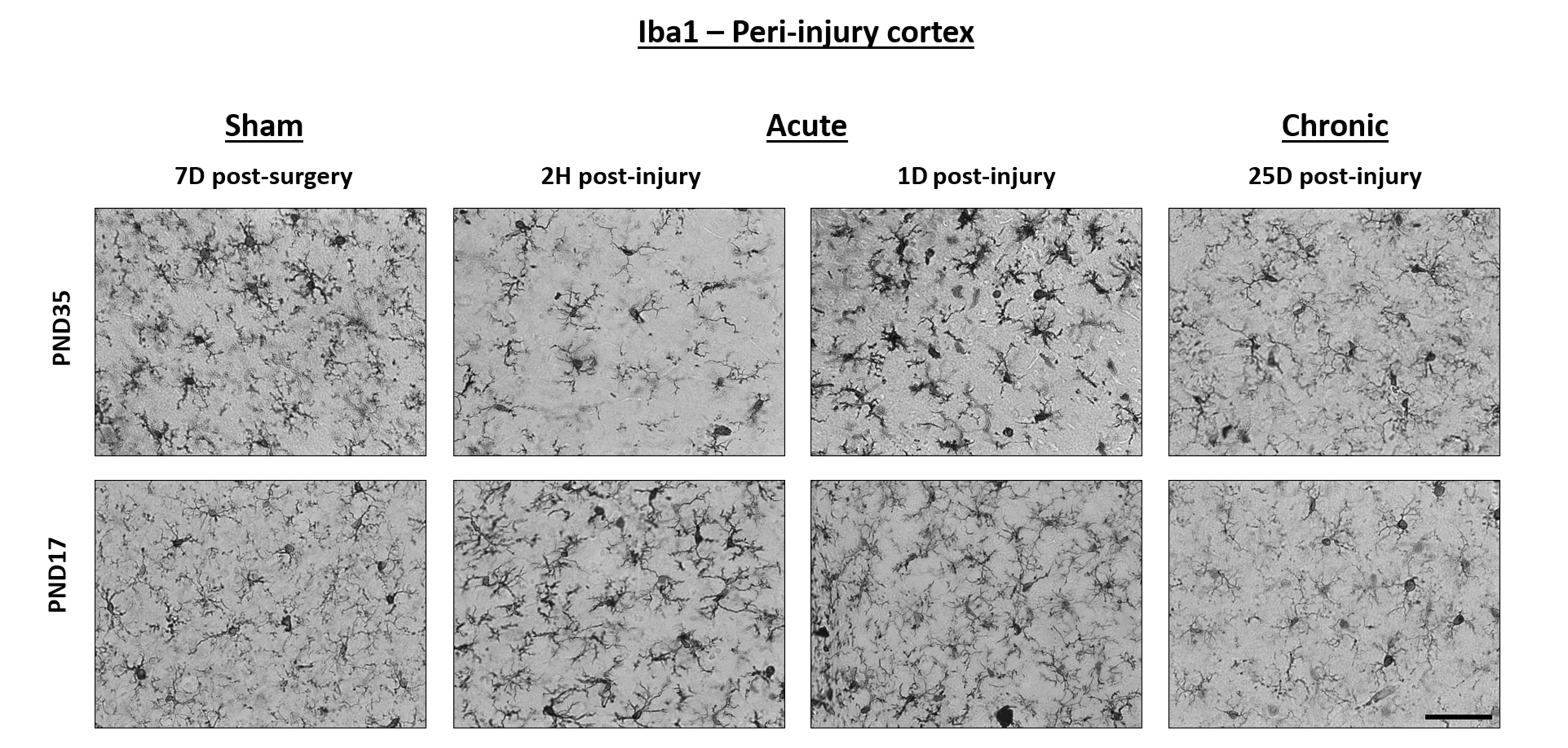

Supplement: Supplementary Figure 1 — Iba1 stained microglia in the peri-injury cortex at 2 h (H), 1 day (D), and 25D post-injury compared to uninjured shams at 7D. All representative images were taken in the peri-injury cortex. Scale bars = 50 μm (sham n = 39, TBI n = 35). [file Image_1.tif]

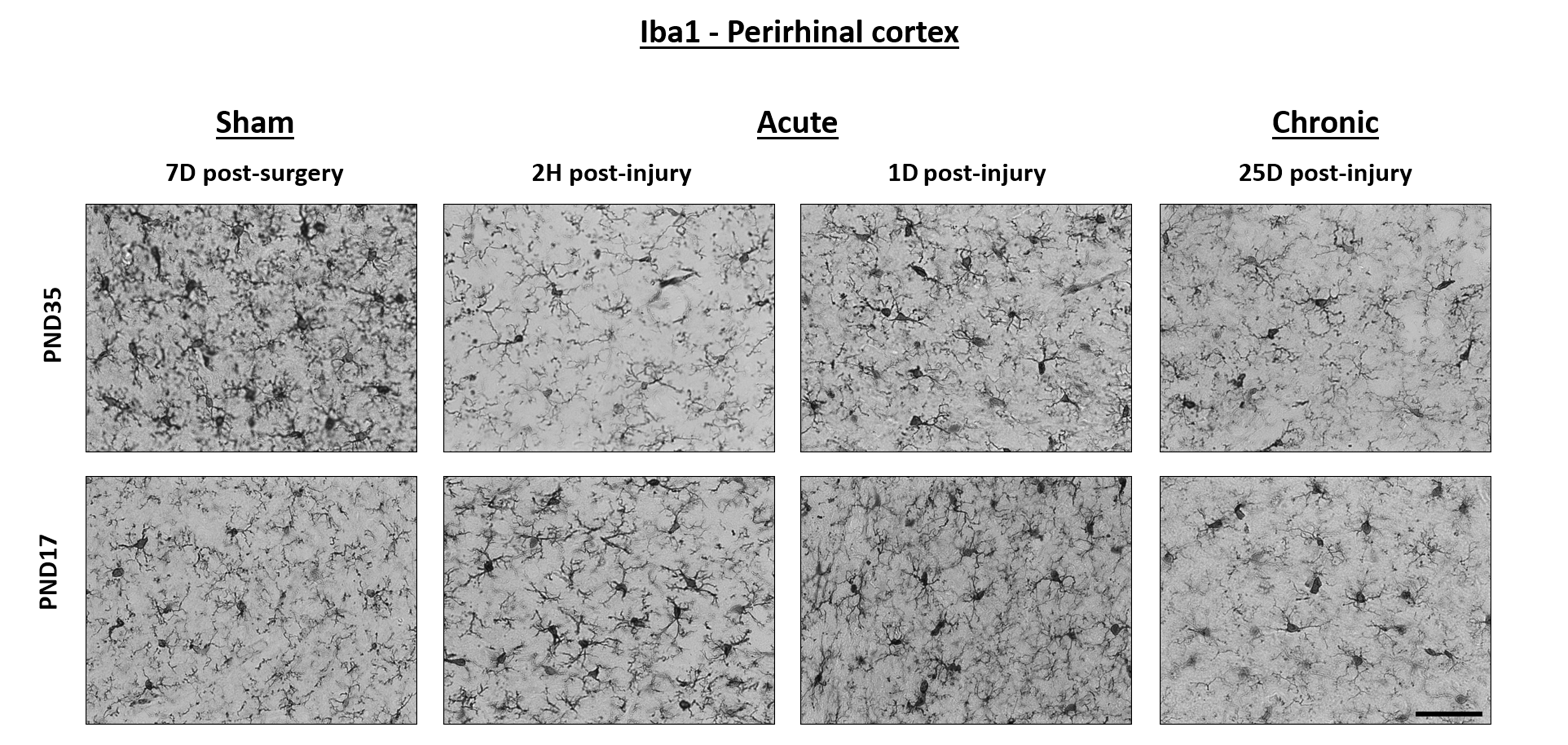

Supplement: Supplementary Figure 2 — Iba1 stained microglia in the perirhinal cortex at 2 h (H), 1 day (D), and 25D post-injury compared to uninjured shams at 7D. All representative images were taken in the perirhinal cortex. Scale bars = 50 μm (sham n = 39, TBI n = 35). [file Image_2.tif]

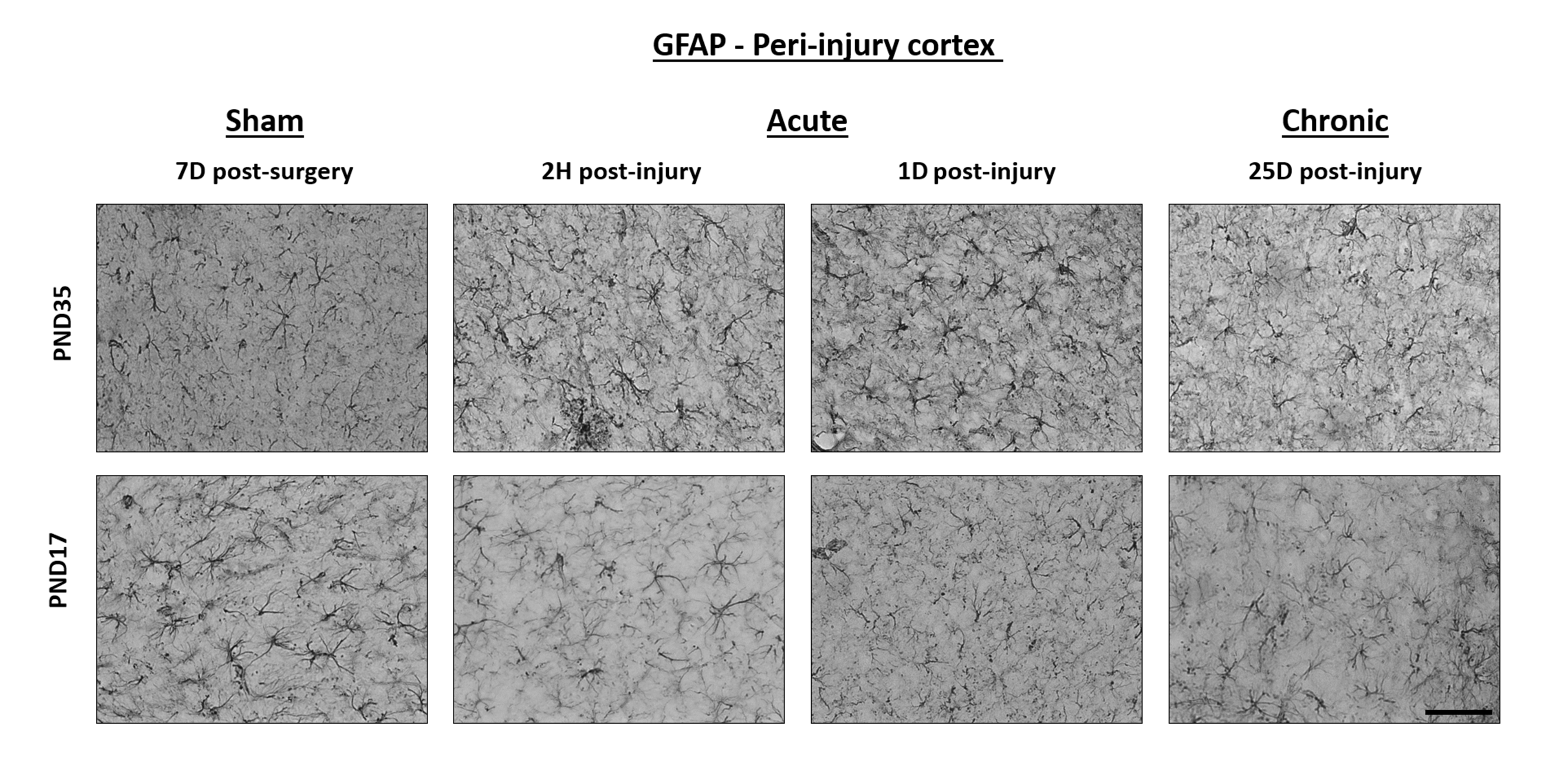

Supplement: Supplementary Figure 3 — GFAP stained microglia in the peri-injury cortex at 2 h (H), 1 day (D), and 25D post-injury compared to uninjured shams at 7D. All representative images were taken in the peri-injury cortex. Scale bars = 50 μm (sham n = 39, TBI n = 35). [file Image_3.tif]

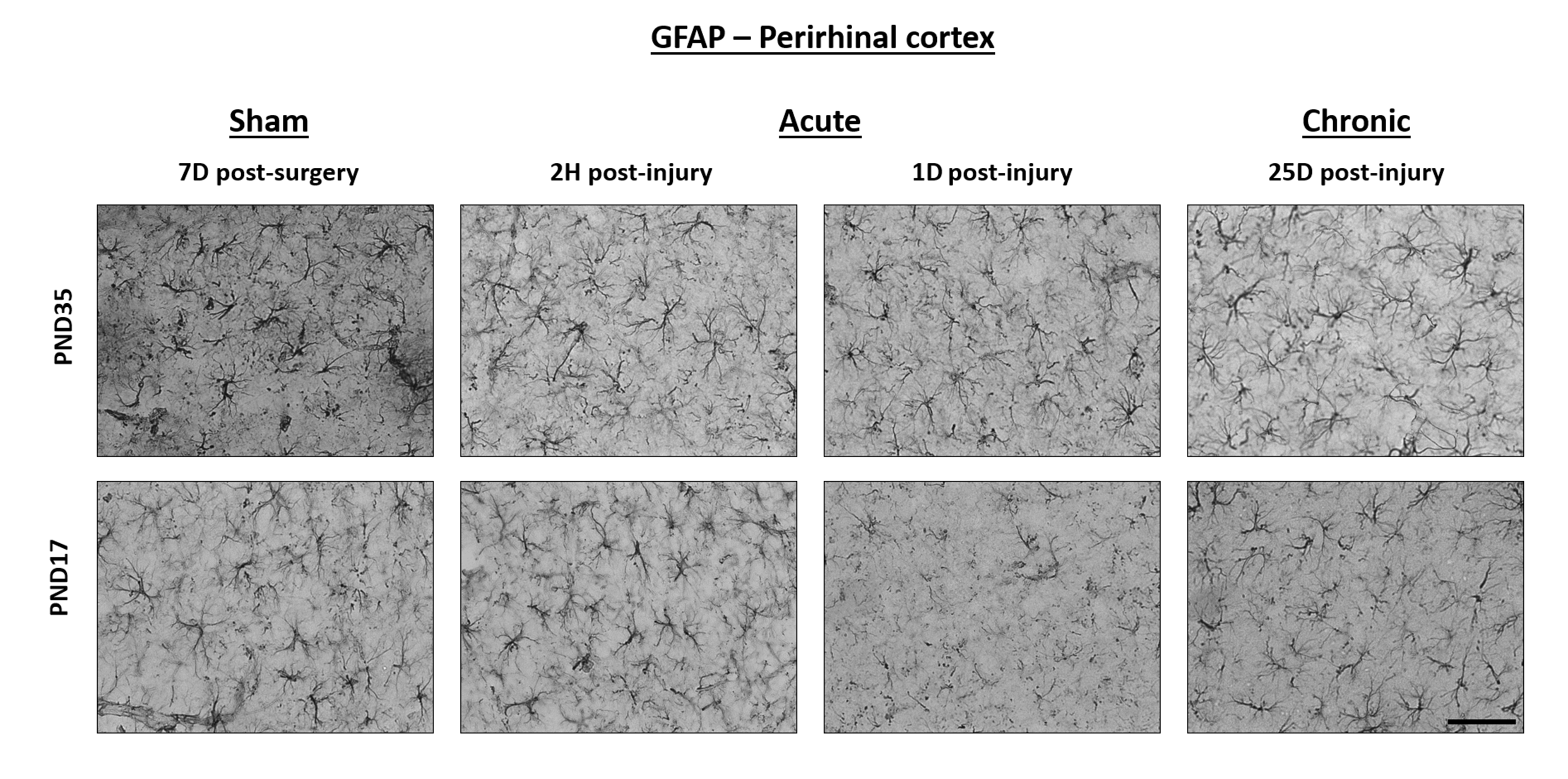

Supplement: Supplementary Figure 4 — GFAP stained microglia perirhinal cortex at 2 h (H), 1 day (D), and 25D post-injury compared to uninjured shams at 7D. All representative images were taken in the perirhinal cortex. Scale bars = 50 μm (sham n = 39, TBI n = 35). [file Image_4.tif]
